# *JXB* at SEB Florence 2018

**DOI:** 10.1093/jxb/ery218

**Published:** 2018-06-13

**Authors:** Christine Raines, Jonathan Ingram

**Affiliations:** 1 Department of Biological Sciences, University of Essex, Colchester, UK; 2 Bailrigg House, Lancaster University, Lancaster, UK

**Keywords:** Climate change, CO_2_-concentrating mechanisms, epigenetics, morphogenesis, plant biotechnology, plant reproduction, plant temperature responses, root architecture

**SEB Annual Meetings cover a diverse range of plant, animal and cell biology, and that diversity extends within each section – as scientists we are all specialists, but we all deeply value the wider connections between disciplines that are a vital part of the organization. That range and ethos is echoed in the plant science published in *Journal of Experimental Botany* (*JXB*) every month, and this year we have put together a virtual issue of the journal both to celebrate our approach and increase awareness of how the content is developing.**

The articles selected follow the plant sessions at the SEB meeting and also include those from the preceding satellite meeting, ‘Advances in Plant Reproduction – from Gametes to Seeds’. A number of papers in the issue cover fascinating aspects of pollination and reproductive investment, including the effects of temperature on pollination in faba bean (*Vicia faba*), which produces a move from selfing to outcrossing in this significant crop (Bishop *et al.*, 2017; Stoddard, 2017), and the mechanism by which wheat flowers open when self-pollination fails, facilitating cross-pollination (Dixon *et al.*, 2017; Okada *et al.*, 2017). These findings are important in understanding fundamental mechanisms and moreover are directly relevant for breeders, and in both cases their wider importance is highlighted and further explored in the accompanying Insight articles by other research scientists. Insights in the eXtra Botany section, a part of our journal since 2016 (Raines, 2016), provide readers with accessible information beyond their specialism and highlight links between disciplines – as with the diverse sessions at the SEB meetings.

Continuing the theme of plant reproduction, Staedler *et al.* (2017) used a new approach with computed tomography-based tools to examine reproductive investment in orchids. The high-resolution 3D imaging of flowers with almost no specimen destruction was used to count the pollen grains and ovules at two different scales in ‘deceptive’ and ‘rewarding’ species. This is interesting not just in the specific situation, understanding the balance between species types and their effects on pollinators, but also in the much wider value of the methodology in counting high-contrast objects within tissues, an aspect covered further by Legland *et al.* (2017). Related reviews in the issue cover the complex biomechanics of seed germination (Steinbrecher and Leubner-Metzger, 2017) and parthenocarpy, i.e. fruit set without fertilization (Joldersma and Liu, 2018; see also the Flowering Newsletter and Blog at floweringhighlights.org).

Moving to broad topics in the SEB meeting, these include the genome/genomics and environmental impacts on epigenetic memory (see Rodriguez-Granados *et al.*, 2016; Shen *et al.*, 2016; Nakamura and Hennig, 2017; Wang and Köhler, 2017; Yolcu *et al.*, 2018); the shaping of root architecture (Amtmann and Shahzad, 2017; Walch-Liu *et al.*, 2017; Du and Scheres, 2018); morphogenesis in non-flowering plants (Peguero-Pina *et al.*, 2017; Veromann-Jürgenson *et al.*, 2017; Thelander *et al.*, 2018); the impact of climate change on forests (Dusenge and Way, 2017; Slot and Way, 2017); general plant temperature responses (Hu *et al.*, 2017); enhancing photosynthesis with CO_2_-concentrating mechanisms (Beardall and Raven, 2017; Ji *et al.*, 2017; Rae *et al.*, 2017); and, finally, plant biotechnology for human health and nutrition (Moses and Goossens, 2017; Poon *et al.*, 2018).

The link with societal issues has always been important in *JXB*, and the work by Poon *et al.* on cyclotides is yet another example of this close connection. It was demonstrated that introduction of a specific asparaginyl endopeptidase gene greatly improves the production of cyclic peptides in a range of plants, including *Nicotiana benthamiana*, as well as crops such as bush bean (*Phaseolus vulgaris*), lettuce and canola. As noted by the authors, cyclotides hold great potential, both as key active molecules themselves and, through their exceptional stability, as facilitators.

## Interpreting the science

Interweaving with regular issues of the journal, our special issues provide a focus on topical areas, and these are led by authoritative Special Issue Editorials, often written by guest editors who have also been involved in organizing the scientific programmes for related conferences. These provide essential context about the overall area of research and act as a useful point of reference for each collection. But increasingly we have encouraged writers to go further than this, taking a view on the state of the field, wider implications and new directions, much as one would take away from participation in the sessions at meetings. In this issue, again linking with major sessions at the SEB meeting, such editorials cover seed development, maturation and germination (Penfield, 2017), the plant hormones auxin (Weijers *et al.*, 2018) and jasmonate (Zhu and Napier, 2017), and plant senescence (Woo *et al.*, 2018). All plant biology links with global environmental issues, but research in *JXB* often makes this connection directly, including in the editorials by Griffiths *et al.* (2017), which advances new evolutionary ideas in the area of carbon concentrating mechanisms in aquatic photosynthesis, and Considine *et al.* (2017), which sets out the need for crop improvement programmes to re-focus on legumes in coming years, addressing the challenge of food security and climate change (Considine *et al.*, 2017).

Prestigious Darwin reviews in *JXB* have provided readers with in-depth accounts of diverse subjects for many years, and those selected for this issue provide coverage ranging from the origins of multicellularity (Niklas *et al.*, 2018) to trace metal metabolism (Andresen *et al.*, 2018), seed germination (Steinbrecher and Leubner-Metzger, 2017) to control of leaf angle (Mantilla-Perez and Fernandez, 2017) and leaf hydraulic decline during dehydration (Scoffoni and Sack, 2017). Sweeping through these is the link between an understanding of basic plant function and effective breeding programmes for a rising population, and the review by Xu *et al.* (2017) on the enhancement of genetic gain, i.e. the increase in performance that can be achieved each year, provides an outstanding, detailed analysis.

## Experimental approaches

Stutz *et al.* (2017) used a ^13^C-labelling technique to examine the complexity of xylem-originating CO_2_ and its effect on measured leaf respiration in trees. The dilemma, so important in understanding carbon allocation and cycling, is further highlighted and explained by Gessler (2017). Such rigorous experimental analysis – with significant implications for our understanding – is another characteristic of the *JXB* approach. The Viewpoint by Dietz (2017) similarly tackles previous experimental limitations, with the focus here on ensuring that subcellular localization is properly considered in investigating the metabolome. Such thought-provoking, short articles in the eXtra Botany section of the journal are welcome and also provide the opportunity to tackle controversial areas (e.g. Blum, 2016; Maron *et al.*, 2016; Shabala, 2017).

## Community support

We are proud of the link between the SEB and *JXB* (it is fully owned by the society), and the cross-disciplinary approach to science exemplified by the SEB Annual Meeting and the journal is clear from the themes and articles explored here. The journal is also pleased to be able to provide direct sponsorship for the satellite meeting ‘Advances in Plant Reproduction – from Gametes to Seeds’ and the session covering morphogenesis in non-flowering plants.

High-impact science and excellent quality metrics, underpinned by an outstanding editorial board, will always be critical for *JXB*. But as a community-led journal other aspects including free open access publication for corresponding authors of subscribing institutions, any reasonable format for initial submission, free data archiving with Dryad Digital Repository (up to 20 GB) and full integration with the BioRxiv preprint server are also given a very high priority. This year publication on acceptance has been a popular new addition ensuring that papers are available at the earliest possible opportunity. We also undertake the highest standards of quality assessment, with friendly and supportive staff making the publication process as efficient as possible.

As always, comments are very welcome – just email the editorial office at j.exp.bot@lancaster.ac.uk with any feedback and/or questions.
